# Is the emergence of life and of agency expected?

**DOI:** 10.1098/rstb.2024.0283

**Published:** 2025-10-02

**Authors:** Stuart Kauffman, Andrea Roli

**Affiliations:** ^1^ Institute for Systems Biology, Seattle, WA 98109-5263, USA; ^2^ Department of Computer Science and Engineering (DISI), Alma Mater Studiorum Università di Bologna, Cesena, Italy

**Keywords:** collectively autocatalytic sets, constraint closure, agency, chiral asymmetry, nested Kantian wholes, first-order phase transition

## Abstract

We present an integrated and testable theory for the spontaneous emergence of life up to the prokaryote with template replication and coding. Collectively autocatalytic small-molecule sets, DNA sets, RNA sets and peptide sets have been discovered or created. Reliable theory supports the claim that such systems can emerge as a first-order phase transition. Such sets constitute Kantian wholes: the whole exists for and by means of the parts. We propose that the earliest life began with small-molecule collectively autocatalytic sets as first-order Kantian wholes. These merged with two other first-order Kantian wholes—peptide and RNA autocatalytic sets—to form a third-order Kantian whole. The autocatalytic, small-molecule set coevolved to become the metabolism of the entire system. The peptide and RNA collectively autocatalytic sets ultimately coevolved to template replication, coding and the ribosome. The same peptide–RNA coevolution may have broken chiral symmetry. Collectively autocatalytic sets achieve *constraint closure*. Thermodynamic work is the constrained release of energy into a few degrees of freedom. In constraint-closed systems, a set of boundary condition constraints on the release of energy, [A,B,C], constrains that release in a set of non-equilibrium processes, [1,2,3], to construct the very same set of boundary condition constraints, [A,B,C]. Cells literally construct specifically themselves. Because constraint-closed systems carry out thermodynamic work cycles, they constitute molecular autonomous agents that are able to sense, orient, decide and act in their worlds. These theories overlap and unite with the RNA world hypothesis.

This article is part of the theme issue ‘Origins of life: the possible and the actual.

## 1. Introduction

For the past four decades, the *RNA world hypothesis* has dominated work on the origin of life. Our purpose is to explore whether an alternative view, *collectively autocatalytic sets*, may now warrant serious research efforts. The two views may overlap in useful ways.

Robertson & Joyce [1] summarized the three central tenets of the RNA world hypothesis:

(i) at some time in the evolution of life, genetic continuity was assured by the replication of RNA;

(ii) Watson–Crick base pairing was the key to replication;

(iii) genetically encoded proteins were not involved as catalysts.

These authors point out that ‘RNA World hypotheses differ about life that may have preceded the RNA World, about the metabolic complexity of the RNA World, and about the role of small molecule co-factors, possibly including peptides, in the chemistry of the RNA World’ [1].

Nobel Laureate Walter Gilbert proposed the RNA world hypothesis in 1986 [2]. Work over the years has focused on attempts to create template-replicating RNA or RNA-like polymers without enzymes [3–5]. Recently, Wachowius and Holliger, using *in vitro* evolution, have obtained an RNA sequence able to act as a polymerase and template to replicate several hundred nucleotides [6].

## 2. Metabolism first: collectively autocatalytic sets

The RNA world states its first tenet: ‘Genetic continuity was assured by the replication of RNA’. It is of major importance that template replication is not the only chemical means to ensure continuity. Persistently reproducing collectively autocatalytic chemical reaction sets affords a different means for chemical reproduction and temporal continuity. Such systems have been proposed for five decades, and DNA, RNA, peptide and lipid collectively autocatalytic sets have been produced experimentally [7–16].

### (a) Collectively autocatalytic systems without catalysts

We distinguish two senses of ‘collective autocatalysis’. In the first, there are no ‘catalysts’, rather, reaction cycles themselves constitute the catalysts. In the other sense, there are catalysts in the system, as discussed below.

Consider a hypothetical open chemical reaction network: A+B→2C, C+D→2E, E+F→2A. There is a reaction cycle: A→C→E→A. If A, C and E are initially in the reaction system, and B, D and/or F are supplied exogenously, the abundance of A, C and E will increase autocatalytically. The reaction cycle itself is the catalyst.

**Figure 1.**
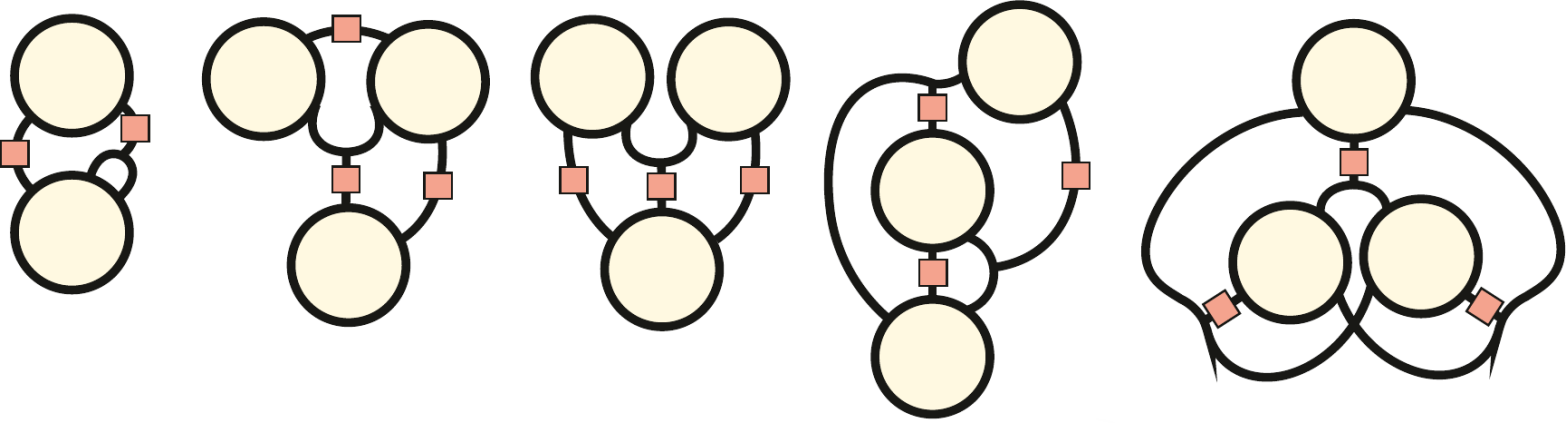
The five collectively autocatalytic motifs. Derived from [17].

Recently, Nghe and collaborators have demonstrated that there are only five specific ‘collectively autocatalytic motifs’ [17] (see figure 1). These five motifs differ in their stability in open reaction systems, increasing from 1 to 5.

Increasing grounds exist to think that chemical reaction cycles are abundant in chemistry space [18]. Wolos and colleagues in 2020 studied ‘Synthetic connectivity, emergence, and self-regeneration in the network of prebiotic chemistry’ [18]. They report an abundance of chemical reaction cycles. These cycles are autocatalytic and hetero-catalytic. If each cycle can act as a catalyst, then mutually linked cycles can act as collectively autocatalytic sets where the cycles themselves are the catalysts.

### (b) Collectively autocatalytic sets with catalysts

**Figure 2.**
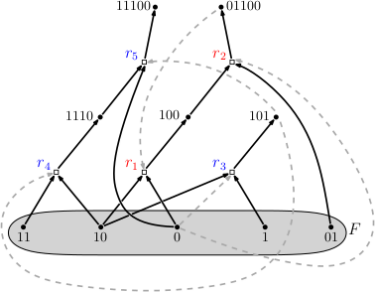
A simple collectively autocatalytic set. The model molecules are bit strings acting as substrates and products of reactions. Black solid arrows are drawn from the dots representing substrates of a reaction to a box representing the reaction. Black solid arrows are drawn from the reaction box to the dots representing the products of the reactions. The actual direction of flow of the reaction depends upon displacement from equilibrium. Dashed lines from dots representing molecules to the boxes representing reactions depict which molecules catalyse which reactions. The exogenously supplied food set of monomers and dimers is shown in the grey oval. Derived from [19].

**Figure 3.**
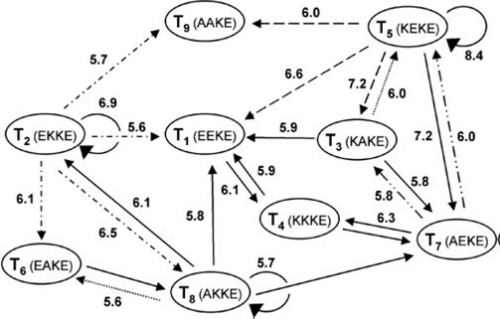
The nine-peptide collectively autocatalytic set discussed in [15]. The ovals show the molecules, and the arrows show the transitions among the molecules and the relative rates.

Figure 2 shows a simple hypothetical chemical autocatalytic set. Figure 3 shows Ashkenasy’s nine-peptide collectively autocatalytic set [15].

As noted, collectively autocatalytic DNA, RNA, peptide and lipid systems have been studied for years [7–16]. Are these reasonable candidates for life? For the earliest life?

*Earliest life*. Perhaps the most powerful recent evidence that molecular reproduction can be based on collective autocatalysis is found in two papers [20,21]. The first [20] demonstrated small-molecule collectively autocatalytic sets with no DNA, RNA, protein or lipid polymers. These sets, each with several hundred small molecules and metals, were found in archaea and bacteria from before oxygen was present in the atmosphere. It is deeply interesting that the two small-molecule collectively autocatalytic sets have an intersection set of 175 small molecules and 172 reactions that is itself collectively autocatalytic. This suggests that the precursor to archaea and bacteria utilized this intersection autocatalytic set. If so, this precursor small-molecule collectively autocatalytic set diverged into the sets found in the archaea and in the bacteria. These sets became the basic metabolism of all subsequent life.

**Figure 4.**
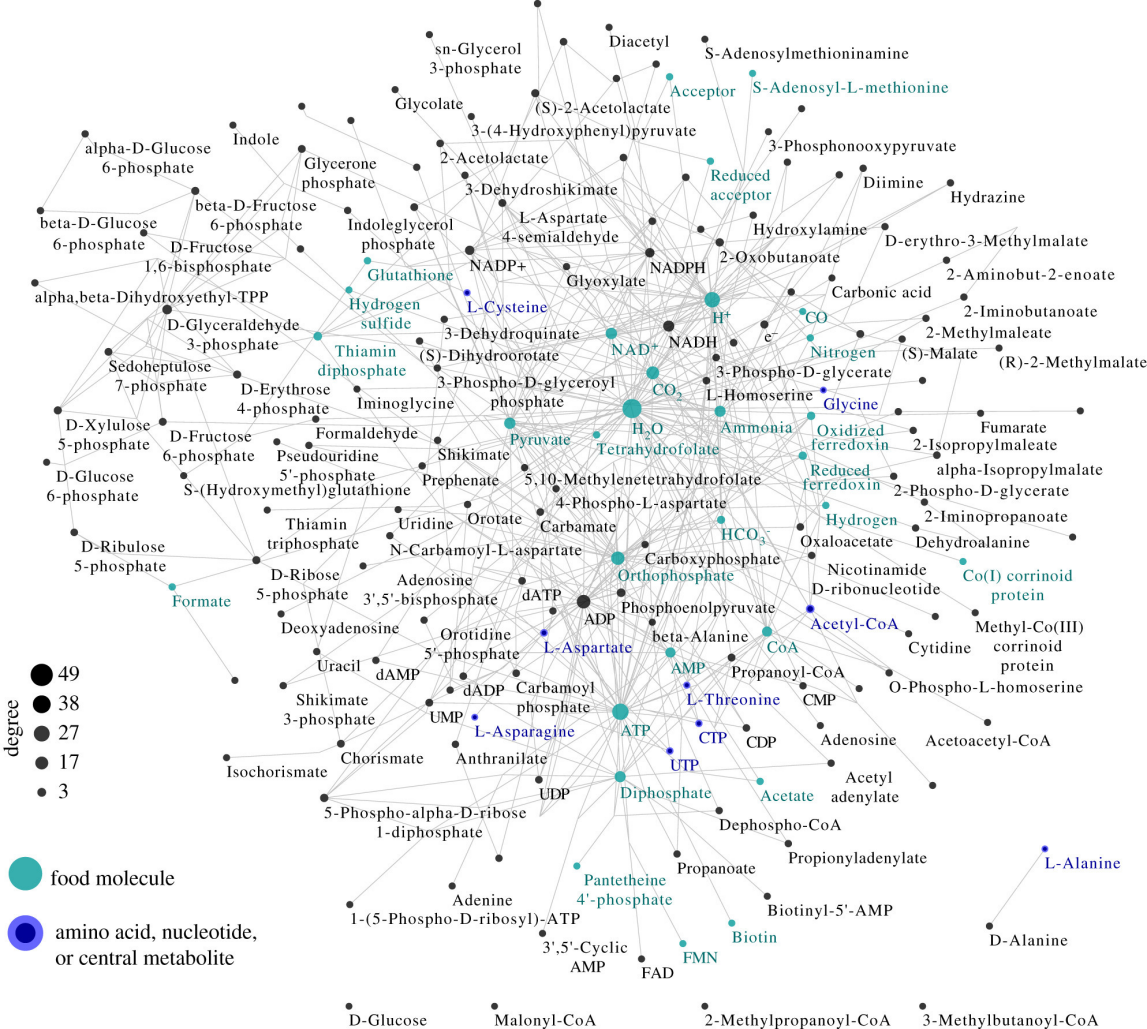
A small-molecule collectively autocatalytic set with no DNA, RNA or peptide polymers in a prokaryote. Similar small-molecule autocatalytic sets are found in all 6700 prokaryotes. Presumably, the phylogeny among these is part of the evolution of metabolism. Derived from [20].

A second paper [21] has shown that all 6700 prokaryotes have small-molecule collectively autocatalytic sets. These sets range in size from a few dozen molecular species and reactions to a few hundred. Presumably, these evolved from the primordial intersection set between archaea and bacteria over 2 billion years ago. An important caveat is that these sets are identified computationally. It has not yet been demonstrated that they reproduce *in vitro* (see figure 4).

It now seems plausible, perhaps highly plausible, that such small-molecule collectively autocatalytic sets were the first form of ‘life’ in the Universe. Such sets typically form a number of amino acids and at least one nucleotide, ATP. In addition, via ATP and NAD, they couple the rudiments of the two major energy systems in living cells. Other sources of energy have been hypothesized as arising from wet–dry cycles [22], or from transmembrane proton and CO_2_ gradients [23].

The existence of small-molecule collectively autocatalytic sets in all 6700 prokaryotes poses a serious challenge to the RNA world hypothesis in which continuity is always maintained by template-replicating molecules. To persist, any such template-replicating RNA would have had to acquire a connected chemical reaction network metabolism leading from simpler chemicals to the needed nucleotide building blocks of the RNA molecule itself, including U, A, C and G.

Here is the challenge: in any such metabolism that evolved to support the RNA template-replicating molecule, there is no reason to think that the metabolism itself would be collectively autocatalytic. On an RNA world hypothesis where continuity is assured by template replication (tenet () above) there is no need for the small-molecule metabolism to be collectively autocatalytic. And to function as a metabolism to support the replicating template, there is no reason for the metabolism itself to be collectively autocatalytic.

The RNA world hypothesis may not itself explain either the origin of life or what existed prior to an RNA world [1]. It seems an attractive hypothesis that small-molecule collectively autocatalytic sets were the earliest form of life on this, and perhaps all life-sustaining planets. This suggests a new union between the RNA world hypothesis and a collectively autocatalytic set view, where such a small-molecule set coevolves to become the metabolism of a later RNA or an RNA–peptide reproducing system.

### (c) The evolution of small-molecule autocatalytic sets

The early archaea and bacteria both have small-molecule collectively autocatalytic sets, with 394 and 292 molecules, respectively, and they share an intersection set of 175 small molecules and 172 reactions that is itself collectively autocatalytic [20]. As noted, presumably the intersection set evolved to the archaea and bacteria sets.

There are two major pathways for such small-molecule collectively autocatalytic sets to evolve:

(i) Collectively autocatalytic sets are typically comprised of one or more *irreducible collectively autocatalytic sets*. Such an irreducible set has the property that if any single reaction is removed from the set, the set is no longer collectively autocatalytic. Irreducible collectively autocatalytic sets, as replicators, can function somewhat as do genes [24]. Such an irreducible collectively autocatalytic set can transition from one to another larger collectively autocatalytic set, rather as do transposons today. Sets can evolve. We can study this evolution of metabolism computationally. It is NP-hard to identify all the irreducible collectively autocatalytic sets in a larger set [19]. However, it is possible to identify many such irreducible sets among those found in 6700 prokaryotes. Let N irreducible sets have been identified. Then each of the small-molecule collectively autocatalytic sets in any one of the 6700 prokaryotes has some specific members among the N. These can be identified. This should reveal patterns in the evolution of metabolism among the prokaryotes.
(ii) The presence of spontaneous reactions with the members of a small-molecule collectively autocatalytic set implies that spontaneous reactions can create small molecules that are not members of the current small-molecule collectively autocatalytic set [25]. If a novel product molecule is created by a spontaneous reaction, and if that product molecule or other molecules in the extant set can catalyse that spontaneous reaction, then the reaction and product molecule are added to the now enlarged small-molecule collectively autocatalytic set.

It may be technically possible to unite microfluidic systems and mass spectrographic instruments to analyse such metabolic evolution. Here, one injects a microvolume droplet of buffer containing a small-molecule collectively autocatalytic set into an oil microfluidic system that cycles the droplet. The droplet can be ‘fed’ by injecting further fluid containing desired input molecules. The droplet can be divided into two droplets that cycle further. One of the two droplets can be fed into a mass spectrograph system to analyse the small molecules in the droplet. The total system has become a flow reactor that can select for increased rates of autocatalysis, and it can also ascertain whether the system has expanded into its chemical adjacent possible. We can study the evolution of metabolisms.^1^

### (d) The emergence of reproduction as a local fluctuation

We have considered two forms of collectively autocatalytic sets: those without catalysts, where the cyclic structures in the reaction networks constitute the catalysts, and collectively autocatalytic sets with true catalysts catalysing the reactions. These two may function together:

(i) Consider a complex reaction network with many autocatalytic and hetero-catalytic cycles. Because the reactions are autocatalytic and the systems are open, some of the members of these auto- and hetero-catalytic cycles may rise to concentrations far above their equilibrium concentrations [18].
(ii) Irreducible small-molecule collectively autocatalytic sets can be comprised of a modest number of molecular species [20,21]. Given high concentrations of some of the different molecules present among the auto- and hetero-catalytic reaction cycles in some locales, these might also happen to comprise at least one irreducible small-molecule collectively autocatalytic set.
(iii) Thus, small-molecule collectively autocatalytic sets might emerge as a local fluctuation. Once formed, such sets may then be able to evolve by exploring their chemical adjacent possible.
(iv) *Molecular reproduction at the level of small molecules could have arisen as a fluctuation*.

### (e) The emergence of collectively autocatalytic polymer sets as a first-order phase transition

The possibility that self-reproducing collectively autocatalytic sets can arise as a *first-order phase transition* has been under investigation since 1971 [7–18,20,21]. Well-grounded theory has shown that in a system with an increasing diversity of molecular species of increasing molecular complexity, at some point a first-order phase transition is reached at which collectively autocatalytic sets spontaneously emerge [7–9,19,26]. Such a phase transition would be the emergence of molecular reproduction. The mathematics underlying this phase transition parallels the first-order phase transition in random graphs as the number of connections between a fixed set of vertices increases. Giant components connecting a large fraction of the vertices in a giant cluster suddenly emerge when the ratio of edges to vertices increases beyond 0.5.

**Figure 5.**
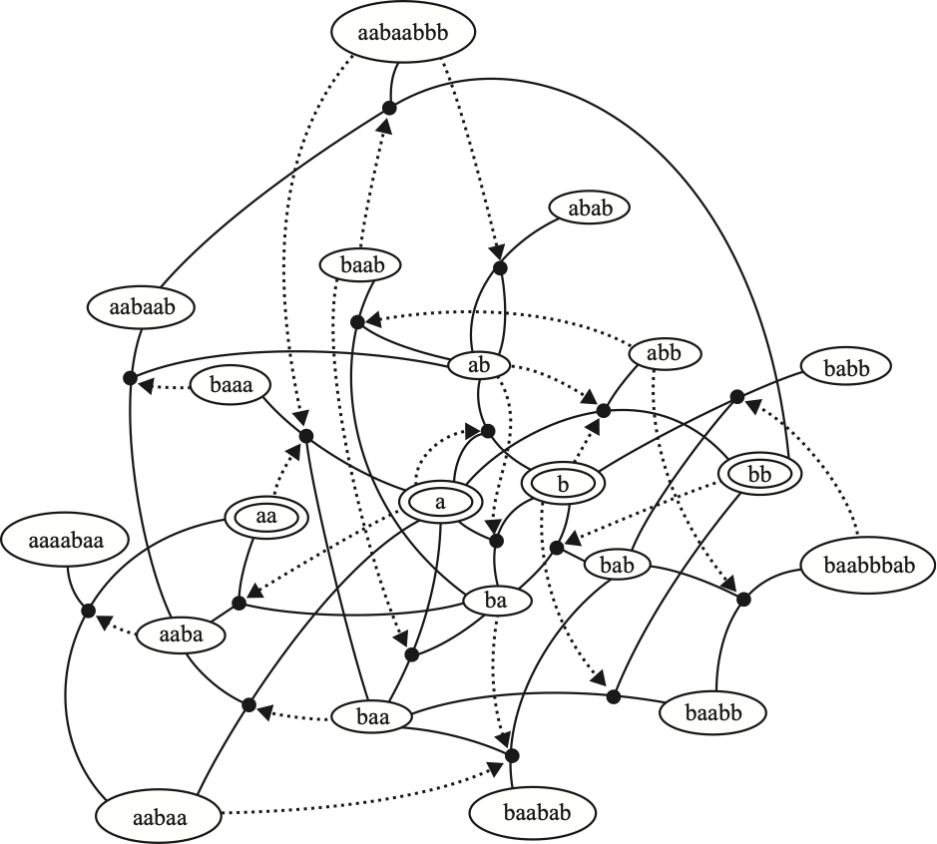
A collectively autocatalytic set of linear polymers derived from [9]. Ovals contain polymers of two monomer types, a and b. Allowed reactions, shown as dots, are cleavage and ligation reactions. A dotted arrow from a molecule oval to a reaction dot indicates that the molecule catalyses that reaction.

With respect to collectively autocatalytic sets, we consider bipartite reaction graphs (see figures 3 and 5). Ovals represent molecules and boxes represent reactions. For each of the molecules and reactions, arrows run from substrate molecules into a reaction box. Arrows run from the reaction box to the product molecules. The directions of arrows do not reflect the direction of thermodynamic flow towards equilibrium.

As the diversity of molecules and atoms per molecule increases, the number of ways to synthesize each of these more complex molecules increases, thus the ratio of reactions, R, to molecules, M, R/M, increases. The analogue of the Erdős–Rényi random graph [27] arises by defining a parameter, $P_{\text{cat}}$, which determines the probability that any molecule catalyses any reaction. The theory randomly assigns, with probability $P_{\text{cat}}$, to each molecule and each reaction a ‘catalytic arrow’, running from the molecule to the reaction it catalyses. The system is now a hyper-bipartite graph.

For a fixed value of $P_{\text{cat}}$, as the number and complexity of molecules increase, so R/M increases, and a first-order phase transition arises at which a collectively autocatalytic set emerges [8,9,26]. This first-order phase transition to collectively autocatalytic sets persists if the distribution of ‘who catalyzes what’ is a power law, uniform or Gaussian [19,26]. The same transition occurs if candidate catalysts must have sub-sequences that ‘recognize’ candidate substrates [25].

Figure 5 shows an example of such an autocatalytic set, derived from [8].

Parallel artificial life theory supports the same results. Years ago, Walter Fontana created ‘algorithmic chemistry’ [28]. Fontana maintained 50 000 Lisp expressions in a model ‘vat’ on his computer. Fontana fed novel Lisp expressions into his vat, and randomly removed Lisp expressions from the vat to sustain the total number of Lisp expressions at 50 000. The system models a flow reactor. Lisp expressions can act on one another to produce new Lisp expressions. By maintaining a constant total number of Lisp expressions in the vat, the system selects for Lisp expressions that increase in abundance. Fontana found, first, Lisp expressions that copied themselves and took over the vat. Further, when he disallowed these and re-ran his experiments, Fontana reliably found the spontaneous emergence of collectively autocatalytic sets of Lisp expressions.

Very recently, Agüera y Arcas *et al.* have extended Fontana’s work with eight different base languages. They found the same spontaneous emergence of collectively autocatalytic sets in seven of the eight [29].

A sensible summary of the theory over the past five decades is that the spontaneous emergence of collectively autocatalytic sets of polymers, RNA, peptides, lipids, or all three, as a first-order phase transition can be expected [7–16,19,25,26,28,29].

## 3. Experiments

If the above is true, and the abiotic universe generated complex molecular and polymer mixtures as found on the Murchison meteorite and elsewhere [30], life is expected, but the actual probability of its emergence and abundance in the Universe are yet to be calculated. More data are needed.

Experiments are needed. These all fundamentally ask: if the diversity and molecular complexity increase for some class of molecules, or mixture of classes, can we detect a phase transition emergence to collectively autocatalytic sets? Such experiments are now feasible. Eva Wollrab and Albrecht Ott [31], have run the Miller–Urey experiment for a month and generated thousands of small molecules as evidenced by mass spectrometry analysis. We now have the capability to generate highly diverse libraries of DNA, RNA and peptides [32]. It should therefore be possible now to test in detail whether collectively autocatalytic sets can emerge.

Macroscopic signatures of the emergence of collective autocatalysis would be highly valuable and may exist. Fontana’s early results with algorithmic chemistry in a ‘flow reactor’ hints at one signature [28]. Fontana found that early in the process, the population of Lisp expressions was highly diverse but the copy number of each Lisp expression was low. After collectively autocatalytic sets emerged, the diversity of Lisp expressions dropped sharply and the copy number of each increased. This seems a macroscopic measure to test on a population of coevolving molecular species in a flow reactor maintained to enforce selection for molecules that increase in abundance faster than the flow exit rate from the reactor.

Agüera y Arcas *et al.* suggested that a higher-order entropic measure of the sequence complexity may be able to detect the transition to collective autocatalysis [29].

Assembly theory also supplies a means to assess whether a polymer sequence might be a sign of life [33]. Perhaps the same measure can detect the emergence of polymers in a collectively autocatalytic set and distinguish them from an earlier phase before collective autocatalysis has emerged in the flow reactor.

Beyond such signatures, detailed analysis will be required to assess which molecules catalyse which reaction in such sets to directly confirm collective autocatalysis.

If these experiments succeed with any single class of molecules, small molecules, peptides, RNA or with any mixtures of these classes, we will begin to have good evidence that molecular reproduction can, indeed, have emerged spontaneously in the Universe. Furthermore, for the first time, we will have the beginnings of quantitative data to assess the conditions for, and hence the probability of, such events in the Universe.

## 4. Breaking chiral symmetry

Chiral asymmetry in the molecules of life is famous. In encoded proteins, all amino acids are levorotatory (L). In polynucleotides in DNA and RNA, the nucleotides are all dextrorotatory (D). The fundamental question is why and how life’s molecules broke chiral symmetry. There is no agreed answer ranging from the weak force to beyond [34].

A well-known theory by Frank in 1953 [35] proposes a form of autocatalysis. Given a racemic chemical reaction with D and L products, if the D products autocatalytically amplify the D version, chiral symmetry will be broken. The same holds if L products autocatalytically amplify L. Even more strongly, if the autocatalysis of D inhibits any autocatalysis of L, and *vice versa*, the system breaks symmetry either to all D or all L.

It is straightforward to generalize this basic idea to collectively autocatalytic sets of chiral polymers such as peptides or RNA. Assume a single testable postulate: homochiral polymers can function better as substrates, products and/or catalysts in reaction networks than can racemic polymers.

This postulate should be testable in racemic and homochiral polymers in collectively autocatalytic sets. For example, consider a homochiral all L peptide autocatalytic set. Substitute a modest fraction of the amino acids with D and test whether, under selection, the system returns to fully homochiral L.

The postulate above links collectively autocatalytic sets to the older theory from 1953. Each homochiral collectively autocatalytic set amplifies itself. In the presence of the other chirality, polymers that are all L and polymers that are all D amino acids will recombine by transpeptidation. This will yield racemic polymers that should be less efficient at any collective autocatalysis. Selection should yield the re-emergence of homochiral, or nearly homochiral autocatalytic sets. If confirmed, we would have a new basis to account for homochirality, with all L among encoded amino acids and all D among genetic polynucleotides. But the converse implication would be that collectively autocatalytic peptide sets and collectively autocatalytic RNA sets must have played a substantial role in the evolution of life on Earth. Such results would be consistent with the theory we develop here.

It is of considerable interest that a recent experiment demonstrates precisely the breaking of chiral symmetry and emergence of chiral molecules, 5-pyrimidyl alkanol, in a simple autocatalytic system [36].

## 5. Kantian wholes, catalytic closure, constraint closure and spatial closure

(a) Living organisms are open, thermodynamic, self-reproducing chemical reaction networks that are Kantian wholes that achieve catalytic closure, constraint closure and are spatially bounded, or enclosed, often by an enclosing lipid membrane.
(b) A *Kantian whole* [37] has the property that the parts exist (in the Universe) for and by means of the whole. You are a Kantian whole. You exist for and by means of your organs—heart and liver and genes. They exist for and by means of being parts of you, the whole.
(c) The molecules in living cells form collectively autocatalytic sets. Each molecule has at least one last step in its formation catalysed by some molecule in the set or in the set of molecules that constitute its exogenous ‘food’ [7–9,19,26,38]. This is clear in Ashkenasy’s nine-peptide collectively autocatalytic set (see figure 3). This is catalytic closure [7–9,19,26,38]. (In reality, some reactions are spontaneous.)
(d) Living cells achieve a newly recognized and powerful property: *constraint closure*.
  (i) Thermodynamic work is the constrained release of energy into a few degrees of freedom [39]. A cannon with powder and a cannonball at its base is an example. The cannon is a boundary condition constraint on the release of energy. When the power explodes, it blasts the ball down the hollow bore of the cannon. Without a boundary condition constraint on the release of energy, no thermodynamic work can be done [39].
  (ii) Thermodynamic work can construct boundary condition entities that can serve as constraints. For example, thermodynamic work was used to construct the cannon [40].
  (iii) In a constraint-closed system [41–43], a set of non-equilibrium processes [1,2,3] and a set of boundary condition constraints [A,B,C] coordinate in such a way that the constraints [A,B,C] constrain the release of energy in the processes [1,2,3], such that the work done constructs the very same set of constraints [A,B,C]. For example, A constrains the release of energy in process 1 to construct a B. B constrains the release of energy in process 2 to construct a C. C constrains the release of energy in process 3 to construct an A. The system literally constructs itself by doing thermodynamic work to construct the boundary conditions that constrain the release of energy to construct the very same boundary conditions. This is entirely new. We construct our automobiles. These have organized constrained releases of energy that do work. Gas explodes, wheels turn. But automobiles do not construct their own constraints on the release of energy. Cells do. Living cells construct themselves [42,43]. Owing to constraint closure, living cells construct *specifically* themselves. The familiar distinction between hardware and software vanishes. Constraint closure is an aspect of classical physics. We have not had the concept before.
  (iv) The union of Kantian whole, catalytic and constraint closure constitutes the mysterious *elan vital* of Bergson [44], here rendered entirely non-mysterious.

## 6. The evolution of prokaryotes

Our aim in this section is to sketch a testable pathway from small-molecule, peptide and RNA collectively autocatalytic sets to the emergence of prokaryotes with template replication and encoded protein synthesis.

The concept of a Kantian whole has enormous power. Given the definition of a Kantian whole, we derive a non-circular definition of the *function* of a part in the whole. *The function of a part is that subset of its causal consequences that sustains the whole*. The function of your heart is to pump blood, not jiggle water in the pericardial sac.

Natural selection acts *directly* on the Kantian whole, and only *indirectly* on its sustaining parts as their functions may improve. Vertebrates with better hearts have more offspring who inherit the better hearts. There is no direct selection on the heart.

Kantian wholes can form *nested Kantian wholes*. Consider a prokaryotic cell to be a first-order Kantian whole. A eukaryotic cell with chloroplasts and mitochondria is a second-order Kantian whole containing the first-order chloroplast and mitochondrion Kantian wholes. A multi-celled organism is a third-order nested Kantian whole. We, with our gut microbiome, are a fourth-order nested Kantian whole. It is of interest to ask whether the entire biosphere is comprised of nested Kantian wholes.

Consider then, early life with three different first-order Kantian wholes: (i) small-molecule collectively autocatalytic sets, (ii) peptide collectively autocatalytic sets, (iii) RNA collectively autocatalytic sets.

Now consider the union of the small-molecule sets and the peptide sets, or of the small-molecule sets and the RNA sets. These each form second-order Kantian wholes. Selection will act directly on this new, *higher*-order Kantian whole and indirectly on its parts. Such selection at the level of the higher-order whole might well select for the coevolution of the autocatalytic small-molecule set to become the metabolism of the small-molecule–peptide set or the small-molecule–RNA set. An immediate advantage of the fact that the metabolism itself is already collectively autocatalytic is that the takeover of catalysis of some of the metabolic reactions by peptides or ribozymes can be piecemeal as the second-order set evolves. Each set helps the other.

A step further might well unite peptide and RNA autocatalytic sets. Autocatalytic RNA sets reproduce subexponentially owing to the difficulty in strand separation of the double-stranded RNA form [45,46]. But if the RNA Watson–Crick strands can each form one or more stem-loops, and if peptides can bind these stems, then the peptides help to destabilize the double-stranded RNA and so help the RNA autocatalytic set to reproduce exponentially. Conversely, if the Watson–Crick strands each have two stem-loops and two peptides bind the two loops of the Watson–Crick strand, then that strand is acting as a ligase to help form a peptide bond between the two peptides that are part of the autocatalytic peptide set [45,46]. The peptide and RNA autocatalytic sets can coevolve. If this can occur, *a new third-order Kantian whole emerges with a metabolic, peptide and RNA collectively autocatalytic system evolving. Selection now acts directly on the third-order Kantian whole, and indirectly on its metabolic, peptide and RNA parts*.

The framework just sketched sets the stage for a plausible pathway for the ultimate evolution of template replication and even coding. Such coevolving third-order nested Kantian wholes may have been evolutionarily stable for some time.

### (a) Template replication

Consider our hypothetical protocell, a third-order Kantian whole embracing metabolic, peptide and RNA autocatalytic sets. Suppose that RNA replication arises by the replication of two or more independent RNA collectively autocatalytic sets. To be concrete, let the polymer lengths of the RNA in each of the two sets be 20 nucleotides. During this replication, the double-stranded form of each of the 20 nucleotide sequences is present and does not readily melt. Let the two 20-*mer*, double-stranded RNA sequences *transiently* stack 3′–5′ end to end. Let the stack fall apart to permit further replication of each of the two independent RNA autocatalytic sets. In this setting, consider that some RNA, or some peptide in the protocell, is able to template replicate all 20 of any one of the four 20-*mers*. This helps the later replication of the relevant RNA autocatalytic set [46].

The suggestion is that an RNA or peptide polymerase can evolve *piecemeal* to help the replication of the separate RNA autocatalytic sets. This affords a potential pathway to the later emergence of an RNA or protein polymerase. At such a stage, replication must transition from the reproduction of independent autocatalytic RNA sets to the replication of the entire RNA genome by the polymerase. Experiments here seem feasible.

### (b) Coding

The same framework may afford a plausible pathway to the evolution of coding. Such a theory builds upon the above, plus the important fact that L amino acids bind their current D anti-codon [47,48]. Thus, just as above, selection for L peptides that help melt the double-stranded D RNA form can coevolve in the peptide–RNA collectively autocatalytic set if the double-stranded D RNA form has one or more stem-loops that the L amino acids or peptides prefer to bind. Conversely, if the D RNA strands have two stem-loops, each of which binds an L peptide of the collectively autocatalytic peptide set, that RNA acts as a ligase to help the peptide set. As discussed in detail elsewhere, this coevolution can lead to polypeptide–polynucleotide co-linearity, and from there to coding [45].

### (c) Chiral asymmetry

But more: if L amino acids and peptides do *not* bind their L RNA anticodons, this hints that the co-evolution of peptide–RNA autocatalytic sets simultaneously broke the racemic symmetry among amino acids to L, and it also broke the racemic symmetry among nucleotides to D. Experiments seem feasible.

The same theory suggests that RNA stem-loops played a role in ligating pairs of peptides or an amino acid and a peptide. Such RNA stem-loop ribozymes may have been the precursors to the ribosome [45]. In parallel, such stem-loop ribozymes could have played a role in the evolution of the two classes of aminoacyl-transferases that charge transfer RNAs [45].

Our hope is that the body of theory and work outlined above sketches a testable pathway for the emergence and early evolution of life from small-molecule collectively autocatalytic sets to nested Kantian wholes comprised of small-molecule autocatalytic, RNA autocatalytic sets and peptide autocatalytic sets that then evolve to full prokaryotes with template replication, coding, and translation, as well as ribosome function. Many steps seem testable.

In a paper expanding upon Fontana’s work described above, Szathmáry, as early as 1995 [49], discusses in detail the emergence and coevolution of collectively autocatalytic systems of different levels. Szathmáry also foresees these issues as testable. Long ago, T. Gánti also considered the coevolution of template-replicating RNA, a metabolism and a bounding lipid membrane [50].

The emergence of higher-order nested Kantian wholes where selection acts at the level of the highest-level whole, and the lower-level wholes are parts of the highest-level whole, describes all cases of the emergence of new units of reproduction, variation and selection. In their seminal book, *The major transitions in evolution* [51], John Maynard Smith and Eörs Szathmáry emphasize that these transitions all concern the emergence of higher-level units of reproduction, variation and selection. If our hypothesis is correct then the evolutionary process of the emergence of ever higher-level units of selection began with the four first-order Kantian wholes: small-molecule collectively autocatalytic sets, peptide collectively autocatalytic sets, RNA collectively autocatalytic sets and lipid autocatalytic sets.

## 7. The emergence of agency

Living cells not only construct themselves, but as open thermodynamic systems, they must ‘eat’ to survive. In general, cells can evolve to ‘eat’ because living cells are nonlinear, dynamical systems with complex dynamical behaviour that enables living cells, receiving inputs from their environment and acting on that environment, to sense and categorize their worlds, orient to relevant features of their worlds, evaluate these as ‘good or bad for me’ and act based on those evaluations. This is the basis of *agency and meaning*. Agency and meaning are immanent in evolving life. The *semantic meaning* is: ‘I get to exist for a while’. The capacity to ‘act’ is immanent in the fact that living cells achieve constraint closure and do thermodynamic work to construct themselves. The same capacity enables cells to do thermodynamic work on their environment. A cell’s action is embodied, enacted, embedded, extended and emotive [40,42,43,52,53].

Cells are molecular autonomous agents, able to reproduce, do one or more thermodynamic work cycles and make one or more decisions, good or bad for me [52,53]. The capacity to learn from the world, categorize it reliably and act reliably may be maximized if the cell, as a nonlinear dynamical system, is dynamically critical, poised at the edge of chaos [54,55]. Good evidence now demonstrates that the genetic networks of many eukaryotic cells are critical [56,57]. Such networks have many distinct dynamical attractors and basins of attraction. Transition among attractors is one means to ‘make a decision’. It will be of interest to test whether Kantian whole autocatalytic sets can evolve to criticality.

## 8. The evolving biosphere is beyond the Newtonian paradigm

It is beyond the scope of this article to discuss what may be its most important point: powerful grounds exist to conclude that the evolution of the biosphere is beyond the Newtonian paradigm and cannot be deduced. We can use no mathematics based on set theory to do so. The evolving biosphere is a propagating, non-deducible construction, not an entailed deduction [42,43,58]. This claim, if true, is the pivot around which science must change. The science of Newton and quantum mechanics requires a prestated phase space for entailing law. The evolving biosphere progressively constructs an evolving phase space that cannot be deduced. The conditions for entailing law fail.

## 9. Conclusion

We have tried to present an integrated and testable theory for the spontaneous emergence of life up to the prokaryote with template replication and coding.

Our theory is based on the successive emergence of small-molecule, RNA and peptide collectively autocatalytic sets. Each is a Kantian whole. We propose that these unite to form a third-order nested Kantian whole in which the small-molecule set becomes the metabolism of the system, while the peptide–RNA sets ultimately evolve to template replication and coding. The same peptide–RNA coevolution may have broken chiral symmetry.

Reliable theory supports the claim that such systems can emerge as a first-order phase transition in sufficiently diverse chemical reaction networks. Alternatively, small-molecule collectively autocatalytic sets may have emerged as a local fluctuation and evolved further.

Collectively autocatalytic sets achieve *constraint closure*. Owing to constraint closure, living cells construct specifically themselves. The familiar distinction between hardware and software vanishes. Because constraint-closed systems carry out thermodynamic work cycles, they constitute molecular autonomous agents that are able to sense, orient, decide and act in their worlds. Agency, behaviour and perhaps mind, evolve. The evolution of the biosphere is a non-deducible propagating construction, not an entailed deduction. These theories may overlap with the RNA world hypothesis in useful ways.

## Data Availability

This article has no additional data.

## Footnotes

^1^
Gonen Ashkenasy, Wim Hordijk, Stuart Kauffman, Niles Lehman, Sijbren Otto, and Philippe Nghe shared in 2011 a small CERN grant to pursue initial phases of these ideas.
